# Single-cell transcriptomic analysis identifies downregulated phosphodiesterase 8B as a novel oncogene in IDH-mutant glioma

**DOI:** 10.3389/fimmu.2024.1427200

**Published:** 2024-06-26

**Authors:** Zongze He, Yu Peng, Duo Wang, Chen Yang, Chengzhi Zhou, Bo Gong, Siyuan Song, Yi Wang

**Affiliations:** ^1^ Department of Neurosurgery, Sichuan Academy of Medical Sciences and Sichuan Provincial People’s Hospital, University of Electronic Science and Technology of China, Chengdu, China; ^2^ Department of Academic Journal, Sichuan Academy of Medical Sciences and Sichuan Provincial People’s Hospital, University of Electronic Science and Technology of China, Chengdu, China; ^3^ Department of Critical Care Medicine, Sichuan Provincial People’s Hospital, School of Medicine, University of Electronic Science and Technology of China, Chengdu, China; ^4^ Department of Health Management, Sichuan Provincial People’s Hospital, University of Electronic Science and Technology of China, Chengdu, Sichuan, China; ^5^ The Key Laboratory for Human Disease Gene Study of Sichuan Province and Institute of Laboratory Medicine, Sichuan Provincial People’s Hospital, University of Electronic Science and Technology of China, Chengdu, Sichuan, China; ^6^ Department of Neuroscience, Baylor College of Medicine, Houston, TX, United States; ^7^ Clinical Immunology Translational Medicine Key Laboratory of Sichuan Province, Center of Organ Transplantation, Sichuan Academy of Medical Science and Sichuan Provincial People’s Hospital, Chengdu, China

**Keywords:** oligoastrocytoma, glioblastoma multiforme, cell-of-origin, metabolism, pathways, clinical significance, phosphodiesterase 8B (PDE8B)

## Abstract

**Introduction:**

Glioma, a prevalent and deadly brain tumor, is marked by significant cellular heterogeneity and metabolic alterations. However, the comprehensive cell-of-origin and metabolic landscape in high-grade (Glioblastoma Multiforme, WHO grade IV) and low-grade (Oligoastrocytoma, WHO grade II) gliomas remains elusive.

**Methods:**

In this study, we undertook single-cell transcriptome sequencing of these glioma grades to elucidate their cellular and metabolic distinctions. Following the identification of cell types, we compared metabolic pathway activities and gene expressions between high-grade and low-grade gliomas.

**Results:**

Notably, astrocytes and oligodendrocyte progenitor cells (OPCs) exhibited the most substantial differences in both metabolic pathways and gene expression, indicative of their distinct origins. The comprehensive analysis identified the most altered metabolic pathways (MCPs) and genes across all cell types, which were further validated against TCGA and CGGA datasets for clinical relevance.

**Discussion:**

Crucially, the metabolic enzyme phosphodiesterase 8B (PDE8B) was found to be exclusively expressed and progressively downregulated in astrocytes and OPCs in higher-grade gliomas. This decreased expression identifies PDE8B as a metabolism-related oncogene in IDH-mutant glioma, marking its dual role as both a protective marker for glioma grading and prognosis and as a facilitator in glioma progression.

## 1 Introduction

Glioma stands as one of the most prevalent and lethal brain tumors, distinguished by significant cellular, genetic, epigenetic, and environmental heterogeneities (1, 2). Despite considerable advancements in diagnostics and therapies including surgery, radiotherapy, chemotherapy, and immunotherapy, gliomas remain incurable and are associated with high mortality rates (3). Biologically, gliomas predominantly originate from neural stem cells (NSCs) (4–8), NSC-derived astrocytes (9) or oligodendrocyte precursor cells (OPCs) (10–13), accounting for about 80% of malignant central nervous system tumors. The World Health Organization (WHO) classifies gliomas into four grades (I–IV) (14), where grades III (anaplastic glioma) and IV (glioblastoma multiforme, GBM) are high-grade (HG), and grade II typically corresponds to low-grade (LG) glioma (15). Understanding the molecular underpinnings across different glioma grades is crucial for identifying novel therapeutic targets.

Altered cellular metabolism is recognized as a hallmark of gliomas (16, 17). In recent decades, research has concentrated on the metabolism of glucose in cancer cells, with targeting the Warburg effect emerging as a novel concept for glioma treatment (18). Additionally, shifts in lipids (19–21), amino acids (22, 23) as well as other metabolites (24–26) metabolisms in gliomas are drawing increased attention, revealing new metabolic genes, pathways, and therapeutic targets (19, 27). Moreover, accumulating evidence underscores the interplay between cellular metabolism and molecular changes in both cancer and immune cells (28). Genetic and epigenetic mutations, such as IDH mutations (27) and H3K27M mutations (29), drive metabolic reprogramming, potentially creating vulnerabilities in glioma cells (30, 31). These metabolic shifts also significantly impact epigenetics, extending beyond the classical mechanisms of tumor pathogenesis (28, 32). Distinct cell-of-origin properties are also implicated in glioma progression, influencing tumor malignancy and drug sensitivity (33), although the comprehensive cell-of-origin and metabolic atlas across high and low-grade gliomas remain unclear. In 2021, the WHO published the fifth edition of the Classification of Tumors of the Central Nervous System, and IDH mutations have become an official glioma type (34). However, in gliomas, IDH mutations are recognizable in >80% of WHO Grade II/III cases, and IDH mutations are also common in secondary GBM, which can even account for 73% of clinical cases (35). Precisely, because of its uniqueness, various IDH mutant-specific targeting strategies are now in a flourishing stage (36). Therefore, we sought to use novel techniques to provide new evidence on the link between IDH mutations and gliomas.

Recent advancements in single-cell sequencing have provided unprecedented resolution in studying glioma. By employing this technology, the cellular architecture and heterogeneity (37–41), along with fate determinants and regulators (37, 42–49) and the tumor microenvironment’s immune cells (8, 50–52), have been explored extensively. Yet, the metabolic landscape of glioma and its clinical relevance remains largely uncharted through single-cell analysis. This study initiates with single-cell transcriptome sequencing of low-grade (Oligoastrocytoma, WHO grade II) and high-grade (Glioblastoma Multiforme, WHO grade IV) glioma samples. We compared metabolic pathway activities and gene expressions in HG versus LG, validating findings with TCGA and CGGA datasets for clinical relevance. Our results highlight phosphodiesterase 8B (PDE8B) as a potential benign prognostic biomarker for glioma, leading us to further investigate its impact on glioma growth through cellular and animal studies. This research delineates metabolic differences between glioma grades and identifies critical genes in glioma metabolism, offering new insights into glioma progression and treatment strategies, with a focus on PDE8B as a pivotal metabolic enzyme.

## 2 Materials and methods

### 2.1 Patient recruitment and sample collections

Patients were recruited at the local hospital’s neurosurgery clinic from October 2023 to January 2024. Before taking part in the study, all individuals were given complete and accurate verbal and written information about it. Subjects who took part in the study provided written informed consent. Following the screening, four primary glioma samples were obtained from two untreated patients, one female with WHO grade IV glioblastoma in the left temporal lobe as high-grade glioma (HG) and one man with WHO grade II oligodendrocyte astrocytoma in the right temporal lobe as low-grade glioma (LG). **Table 1** contains detailed basic clinical and pathological information on two patients who participated in this study.

**Table 1:** Detailed information about two patients who participated in this research.

Sample Types	Gender	Age	First Symptom	Lesion	Surgery Date	Pathological grade	Ki67	KPS score at discharge after surgery
LG	Male	37	Dizziness	Left temporal lobe	2020/7/20	Oligodendrocyte astrocytoma (WHO grade II)	0.15	90
HG	Female	38	headache	Right temporal lobe	2020/9/4	Glioblastoma (WHO grade IV)	0.3	60

### 2.2 Single-cell transcriptomics profiling of glioma samples

Four fresh samples were collected prior to surgery and digested into single-cell suspensions before being examined using droplet-based single-cell transcriptome profiling through the Cell Ranger software pipeline (version 3.1.0) provided by the 10 x Genomics Chromium system. The number of high-quality cells in each sample after Cell Ranger quantitative quality control ranged from 2419 to 10733. Following the removal of low-quality cells such as doublets, multiplets, and apoptotic cells, the final number of cells collected varies from 1606 to 9744, the average number of UMIs (Unique molecular identifiers) in each cell is 7325 to 15424, the average number of genes in each cell is 2424 to 3858, and the average ratio of mitochondrial genes in each cell is 0.0569 0.1190. We used the R package Seurat (version 3.1.1) to process the filtered unique molecular identifier (UMI) count matrix (28). The algorithm provided by Macosko et al. was used to identify the top variable genes across single cells (29). Principal component analysis (PCA) was used in Seurat to reduce dimensionality using the RunPCA function (PC num = 15) (28). We used the FindClusters function to analyze cell groups using graph-based clustering according to their gene expression profiles, the RunTSNE function to display clusters using a 2-dimensional t-distributed stochastic neighbor embedding (t-SNE) method, and the FindAllMarkers function to find marker genes in each cluster in Seurat (28). Then, we used the R package SingleR, an automated annotation method for unbiased scRNA-seq cell type detection, with Human Primary Cell Atlas from Mabbott et al. (53). as the reference transcriptome datasets, to infer the cell types (30, 31, 54). The FindMarkers function in Seurat was used to identify differentially expressed genes (DEGs). The criterion for substantially different expression was established at P.Value = 0.05 and |log2foldchange| > 0.58. The hypergeometric distribution was used to perform GO (Gene Ontology) enrichment and KEGG (Kyoto Encyclopedia of Genes and Genomes pathway enrichment) analyses of DEGs.

### 2.3 Tissue staining

Glioma tissues were fixed in 4% paraformaldehyde (P0099, Shanghai Beyotime Biotechnology Co., Ltd.) for 24 h and dehydrated with gradient alcohol. The sections were embedded in paraffin, stained with hematoxylin (517–28-2, Sigma-Aldrich Corporation) for 5 min. After washed with running water, the sections were differentiated with 1% hydrochloric alcohol for 5–10s. The sections were counterstained with eosin (15086–94-9, Sigma-Aldrich Corporation) for 3 min. After dehydration with ethanol and absolute ethanol (64–17-5, Chengdu Kelong Chemical Co., Ltd.), the slices were observed under a light microscope.

The paraffin section of glioma tissues was deparaffinized and hydrated. The sections were incubated with PDE8B (ARG10811, arigo Biolaboratories Corporation), ABAT (64430, Cell Signaling Technology, Inc.) and ADCY2 (PA5–114701, Thermo Fisher Scientific) primary antibody overnight at 4°C. The sections were then stained with the appropriate HRP-labeled polymer-conjugated secondary antibody (C31460100, Thermo Fisher Scientific) for 60 min. The sections were counterstained with hematoxylin for 3 min. Immunostaining images were captured.

### 2.4 RNA isolation and quantitative real time PCR

Total RNA from tissues and cells was extracted using TRIzol reagent (T9424, Merck Corporation). RNA reverse transcription was performed according to the instructions of the Vazyme kit, Nanjing (R211–01, Nanjing Vazyme Medical Technology Co., Ltd.). Real time PCR was performed using synthetic primers for the corresponding genes. The reaction conditions were first predenaturation at 95 °C for 2 min, followed by 40 cycles of denaturation at 95 °C for 10 s, annealing at 60 °C, and extension for 30 s. GAPDH was used as the reference gene. Relative gene expressions were calculated by the 2^-△△Ct^ method. The primer sequences used were as follows: GAPDH: Forward, 5’-ACAGCCTCAAGATCATCAGC-3’; Reverse, 5’-GGTCATGAGTCCTTCCACGAT-3’. PDE8B: Forward, 5’-ACGCAGGCTTCAACAGGAG-3’; Reverse, 5’-CGTGGTCATCGCTTGTTATTTCT-3’.

### 2.5 Western blotting analysis

The glioma tissues and adjacent tissues were ground with liquid nitrogen, fully lysed with cell lysate. The total protein was extracted. BCA kit (P0010, Shanghai Beyotime Biotechnology Co., Ltd.) was used for protein quantification. A 30uL loading system with 100 ug mass was prepared. Then, the sample was denatured at 95°C for 5 min. The proteins sample were separated by 12% SDS-PAGE gel, and then transferred to the membrane and blocked in 5% skim milk for 1h. Primary antibodies (PDE8B) were incubated at 4°C overnight and secondary antibodies were incubated. Quantitative analysis was performed by Image J software.

### 2.6 Statistical analysis

All of the experiments were performed at least three times. GraphPad Prism 9.0 statistical software was used to analyze the data. Measurement data in line with normal distribution were expressed as mean ± standard deviation. Student’s t-test was used for comparison between two groups, and one-way analysis of variance was used for comparison between multiple groups. *P*<0.05 was considered statically significant.

## 3 Results

### 3.1 Overall metabolism landscape of high grade and low grade glioma

**Figure 1:**
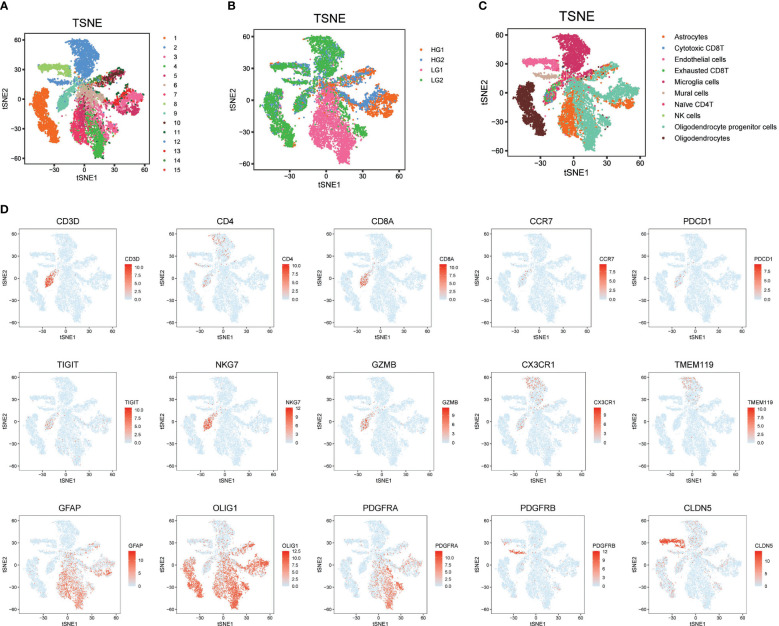
Metabolism prolife and cellular architecture in high and low grade glioma. **(A)** All 15 clusters were generated with dimension reduction; **(B)** T-SNE map showing the distinct metabolism characteristics in HG and LG samples; **(C)** Cell type distribution identified with classical markers in T-SNE map; **(D)** Representative cell markers used in this study for cell type identification.

Initially, to acquire a single-cell expression matrix from high-grade (HG) and low-grade (LG) glioma, two fresh tissue samples per group were utilized. These were clinically and pathologically identified as WHO grade IV Glioblastoma Multiforme and WHO grade II Oligoastrocytoma for the HG and LG groups, respectively. After preprocessing, quality control, and dimension reduction of 1667 metabolic genes in the KEGG pathway database, 15 clusters were generated, as displayed in **Figure 1A**. The t-SNE map revealed distinctly different expression patterns between HG and LG groups, suggesting unique metabolic landscapes for each (**Figure 1B**). Based on the expression specificity of known markers (**Figure 1D**), these 15 clusters were categorized into 10 cell types: Astrocytes, Cytotoxic CD8+ T cells, Endothelial cells, Exhausted CD8+ T cells, Microglia cells, Mural cells, Naïve CD4+ T cells, NK cells, Oligodendrocyte progenitor cells (OPCs), and Oligodendrocytes (**Figure 1C**).

### 3.2 Comparative analysis of metabolic landscape in HG and LG glioma

**Figure 2:**
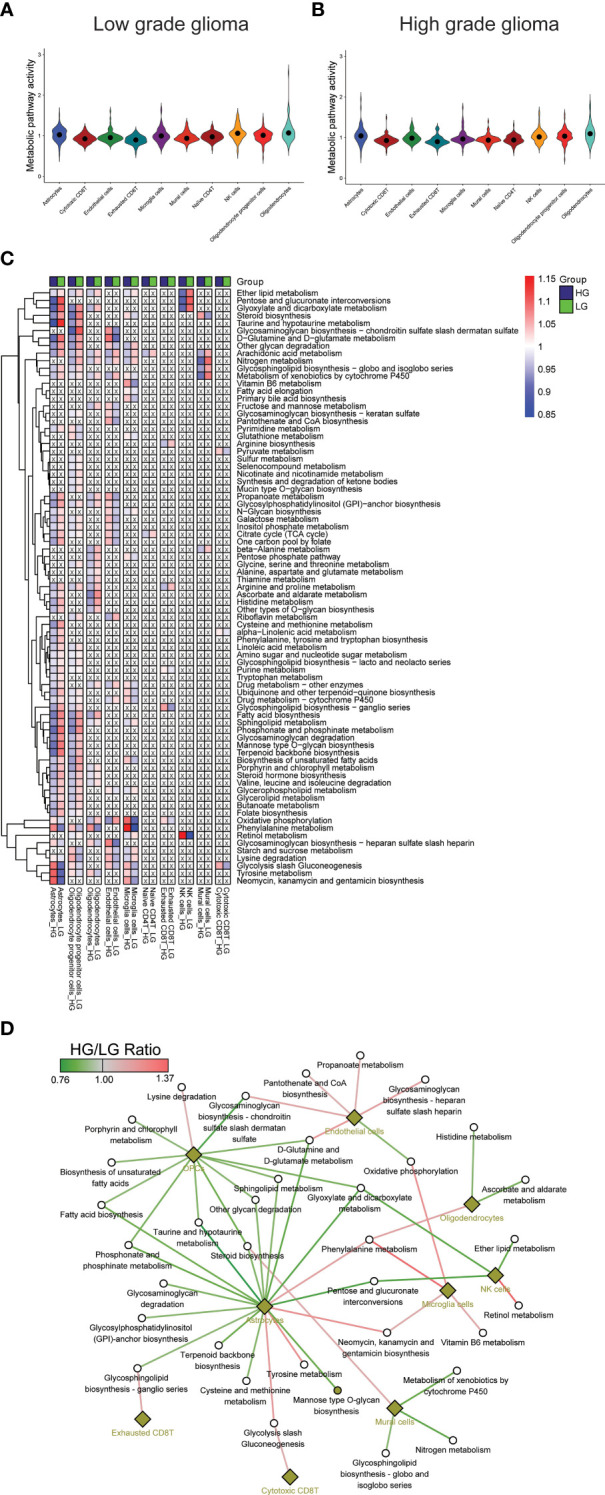
Metabolism pathways comparison in cell types. **(A, B)** Overall metabolism pathway activities in all 10 cell types of both HG and LG glioma; **(C)** Metabolic heatmap showing the differential activities of 79 pathways in all 10 cell types; **(D)** Glioma grade differential network of pathways with corresponding cell types.

The metabolic landscape between HG and LG glioma was further explored by comparing the metabolic pathway activities and gene expressions. Utilizing 85 metabolic pathways and 1667 genes from the KEGG pathway database, as described in the algorithm reported by Xiao et al. (55), pathway activities for the 10 cell types in both HG and LG groups were calculated, with overall pathway activities illustrated in **Figures 2A, B**. Notably, the highest metabolic activity in LG glioma was observed in oligodendrocytes, ranking second in the HG glioma group. Conversely, astrocytes exhibited the highest metabolic activity in HG glioma, suggesting a distinct cell-of-origin basis for oligodendrocytes and astrocytes in LG and HG glioma, respectively.

### 3.3 Identification of most changed metabolic pathways in all cell types

Further analysis compared individual metabolic pathway activities across the 10 cell types in both groups. Metabolically changed pathways (MCPs) was defined as having HG-activity >1 and LG-activity <1, or the reverse (**Supplementary Table 1**). Of all, 79 MCPs were identified, with 6 pathways observed in more than five cell types, including Arachidonic acid metabolism, Metabolism of xenobiotics by cytochrome P450, Glycolysis/Gluconeogenesis, Oxidative phosphorylation, Nitrogen metabolism, and Ether lipid metabolism. Most MCPs were prevalent in astrocytes (55/86) and OPCs (53/86), followed by oligodendrocytes (27/86), microglia cells (26/86), and endothelial cells (25/86), with other cell types showing fewer MCPs (**Figures 2A, B**), aligning partially with the overall metabolic landscape depicted in **Figure 2C**. A stricter criterion was applied to pinpoint the most changed MCPs, setting the pathway activity ratio (HG/LG) at >1.1 or <0.9. As a result, 35 MCPs across 9 cell types were identified (**Supplementary Table 2**, **Figure 2D**). Notably, while most MCPs in astrocytes and OPCs showed decreased activity, endothelial cells and microglia cells displayed increased activity in the HG group. Despite most MCPs exhibiting consistent trends across most cell types, a small number demonstrated converse trends in distinct cell types, such as a decrease in oxidative phosphorylation activity in endothelial cells and an increase in microglial cells.

### 3.4 Clinical significance of metabolism pathways in glioma

**Figure 3:**
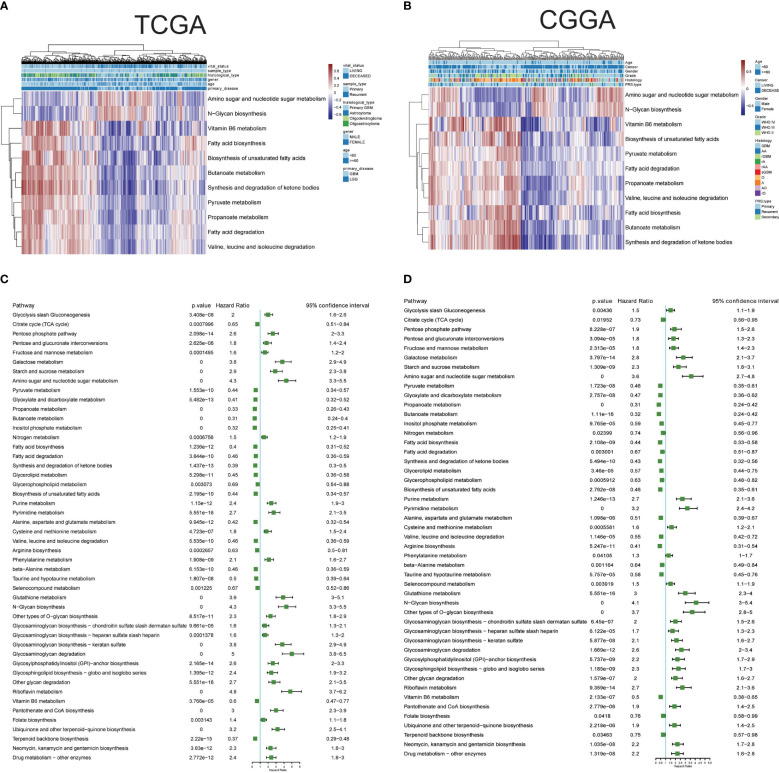
Clinical relevance and prognostic performance of metabolism pathways in glioma. **(A)** Eleven metabolism pathway activities in glioma samples with clinical parameters, in TCGA datasets; **(B)** Eleven metabolism pathway activities in glioma samples with clinical parameters, in CGGA datasets; **(C)** Forest map showing the prognostic performance of metabolism pathway activities in TCGA datasets; **(D)** Forest map showing the prognostic performance of metabolism pathway activities in CGGA datasets.

The single-cell data provided insights into altered metabolic pathway activities in HG and LG glioma. Utilizing bulk sequencing data, TCGA and CGGA datasets were employed to investigate the clinical significance of these pathway activities. Gene set variation analysis (GSVA) of all 85 metabolic pathways was conducted, with the overall activity (GSVA Score) heatmap displayed in **Supplementary Figures 1A, B**. Subsequent statistical analysis of metabolic pathways with clinical parameters used t-tests or one-way ANOVA. All 30 metabolic pathways demonstrated statistical significance (p<0.05) with primary disease, histological type, sample type, and vital status in TCGA datasets, and 21 pathways showed clinical significance (p<0.05) with PRS type, histology, grade, and vital status in the CGGA dataset (**Supplementary Table 3**). The intersection of these pathways in both datasets highlighted 11 pathways with clinical relevance: Amino sugar and nucleotide sugar metabolism, Pyruvate metabolism, Propanoate metabolism, Butanoate metabolism, Fatty acid biosynthesis, Fatty acid degradation, Synthesis and degradation of ketone bodies, Biosynthesis of unsaturated fatty acids, Valine-leucine and isoleucine degradation, N-Glycan biosynthesis, and Vitamin B6 metabolism, as shown in **Figures 3A, B**. Pyruvate metabolism, a well-characterized cell glycolysis and energy metabolism pathway in glioma, showed significant relevance with glioma clinical parameters such as grade, histology, and vital status (**Supplementary Figure 1C**).

### 3.5 Prognostic performance of metabolic pathways

The prognostic performance of these metabolic pathways was analyzed in both CGGA and TCGA datasets, revealing that 59 pathways in the CGGA dataset and 58 in the TCGA dataset exhibited significance for overall survival, with 48 common significant pathways (**Figures 3C, D**, **Supplementary Table 4**). Notably, pathways such as Glycolysis/Gluconeogenesis and the Citrate cycle (TCA cycle) showed either unfavorable or favorable prognostic performance in both datasets. Additional Kaplan-Meier survival plots for another 10 pathways are provided in **Supplementary Figure 2**, covering Carbohydrate metabolism (Pentose phosphate pathway and pyruvate metabolism), Nucleotide metabolism (Purine metabolism and Pyrimidine metabolism), Amino acid metabolism (Cysteine and methionine metabolism, and Glutathione metabolism), Lipid metabolism (Fatty acid biosynthesis and Glycerolipid metabolism), and pathways related to Selenocompound metabolism and Vitamin B6 metabolism. Collectively, these results underscore the clinical significance of metabolic pathways in glioma.

### 3.6 Clinical investigation of astrocytes and OPCs metabolic DEGs in glioma

**Figure 4:**
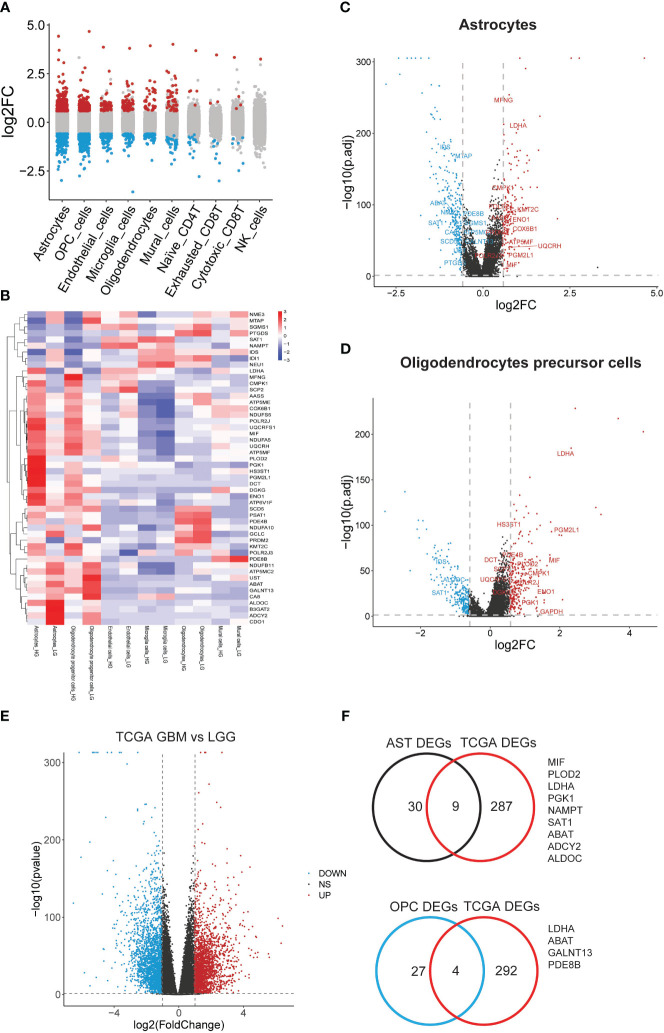
Significant differential metabolic genes in single cells and validated with TCGA datasets. **(A)** Differentially expressed genes in all cell types were acquired by comparing the HG and LG groups; **(B)** Metabolism gene expression heatmap in most changed 6 cell types; **(C)** Metabolism genes with differential expression in astrocytes; **(D)** Metabolism genes with differential expression in PCs were shown in volcano plot and labeled; **(E)** Significant deferential genes were acquired by comparing GBM with LGG samples, shown with volcano plot; **(F)** Common deregulated genes in astrocytes and OPCs were cross-validated with TCGA datasets.

**Figure 5:**
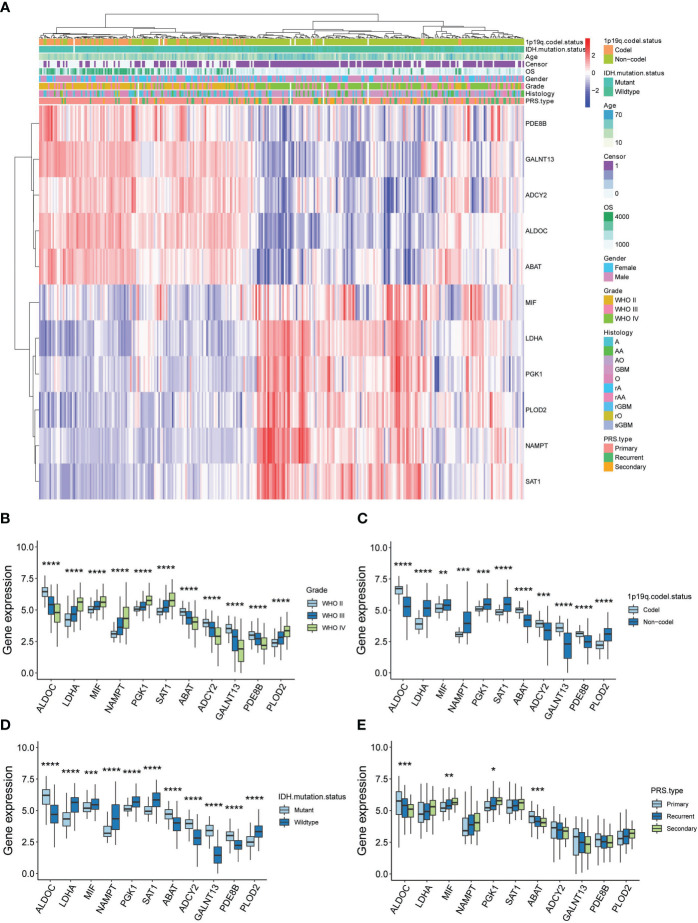
Clinical validation of 11 metabolic genes in CGGA datasets. **(A)** Expression-clinical parameters heatmap of 11 metabolic genes in CGGA datasets; **(B)** Expression of the 11 metabolic genes in WHO grade II, III and IV glioma samples of CGGA datasets; **(C)** Expression of the 11 metabolic genes in 1p/19q co-deletion glioma samples of CGGA datasets; **(D)** Expression of the 11 metabolic genes in IDH wide-type (WT) and mutation glioma samples of CGGA datasets; **(E)** Expression of the 11 metabolic genes in PRS type (Primary, recurrent and secondary) glioma samples of CGGA datasets. *P<0.05, **P<0.01, ***P<0.001.

In a further step, the gene expression profiles between HG and LG groups were compared, leading to the identification of differential genes (DEGs) across 10 cell types (**Supplementary Table 5**). Astrocytes and OPCs exhibited the highest number of DEGs, followed by endothelial cells, microglia, oligodendrocytes, and mural cells (**Figure 4A**). The expression of metabolic DEGs in these six cell types is depicted in **Figure 4B**. Given the prevalence of metabolic DEGs and MCPs in astrocytes and OPCs, these cell types were selected for detailed analysis. The metabolic DEGs in astrocytes and OPCs are presented in volcano plots (**Figures 4C, D**, respectively). These DEGs were also cross-validated with those identified in the TCGA dataset comparing glioblastoma multiforme (GBM) with low-grade glioma (LGG) (**Figure 4E**). Consequently, all 9 and 4 common metabolic DEGs were found in astrocytes and OPCs, respectively, including LDHA and ABAT in both cell types (**Figure 4F**). Thus, attention was focused on these 11 genes (t-SNE map in **Supplementary Figure 3**), and their significance with clinical parameters was investigated in CGGA glioma datasets (**Figure 5A**). Similar to the TCGA datasets, all 11 genes exhibited WHO grade-dependent expression in glioma samples, with 6 upregulated (LDHA, MIF, NAMPT, PGK1, SAT1, and PLOD2) and 5 downregulated genes (ALDOC, ABAT, ADCY2, GALNT13, and PDE8B) (**Figure 5B**). Furthermore, all 11 genes also showed a significant correlation with 1p/19q co-deletion and IDH mutation status (**Figures 5C, D**), with expression trends consistent with WHO grades. Regarding the primary-recurrent-secondary (PRS) type, ALDOC and ABAT exhibited decreased expression, while MIF and PGK1 showed increased expression in recurrent and secondary glioma groups (**Figure 5E**).

### 3.7 Prognostic significance of the 11 metabolic genes

**Figure 6:**
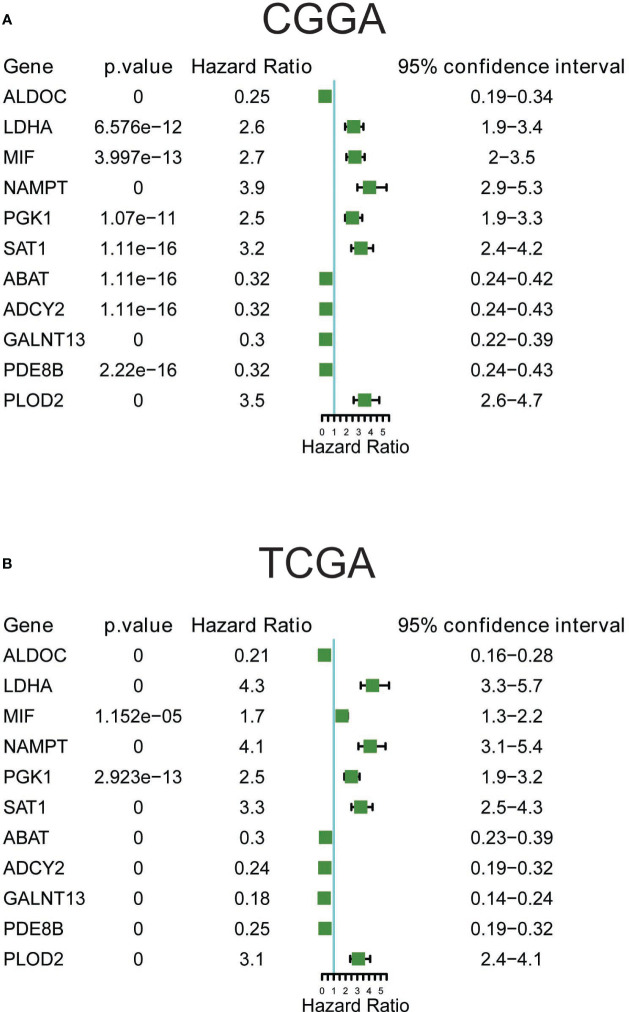
Prognostic significance of the 11 metabolic genes in CGGA **(A)** and TCGA **(B)** datasets.

The prognostic significance of these 11 metabolic genes was further validated using both CGGA (**Figure 6A**) and TCGA (**Figure 6B**) datasets. Consistent with the changes in expression, patients with higher expression of LDHA, MIF, NAMPT, PGK1, SAT1, and PLOD2, and lower expression of ALDOC, ABAT, ADCY2, GALNT13, and PDE8B showed poorer survival rates in all glioma samples (**Supplementary Figure 4**). Additionally, the prognostic significance of these genes in LG and HG was analyzed, and most genes demonstrated consistently good performance in the LG and HG groups in CGGA datasets (**Supplementary Figure 5**). For TCGA datasets, the most significant genes were observed in low-grade groups, such as the favorable performance of ABAT, ADCY2, ALDOC, PDE8B, and GALNT13, as well as the unfavorable performance of LDHA, NAMPT, PLOD2, and SAT1, suggesting prognostic prediction performance of these 11 genes in glioma, particularly in low-grade glioma patients.

### 3.8 PDE8B expression in astrocytes and OPCs and its validation in glioma tissues cohort

**Figure 7:**
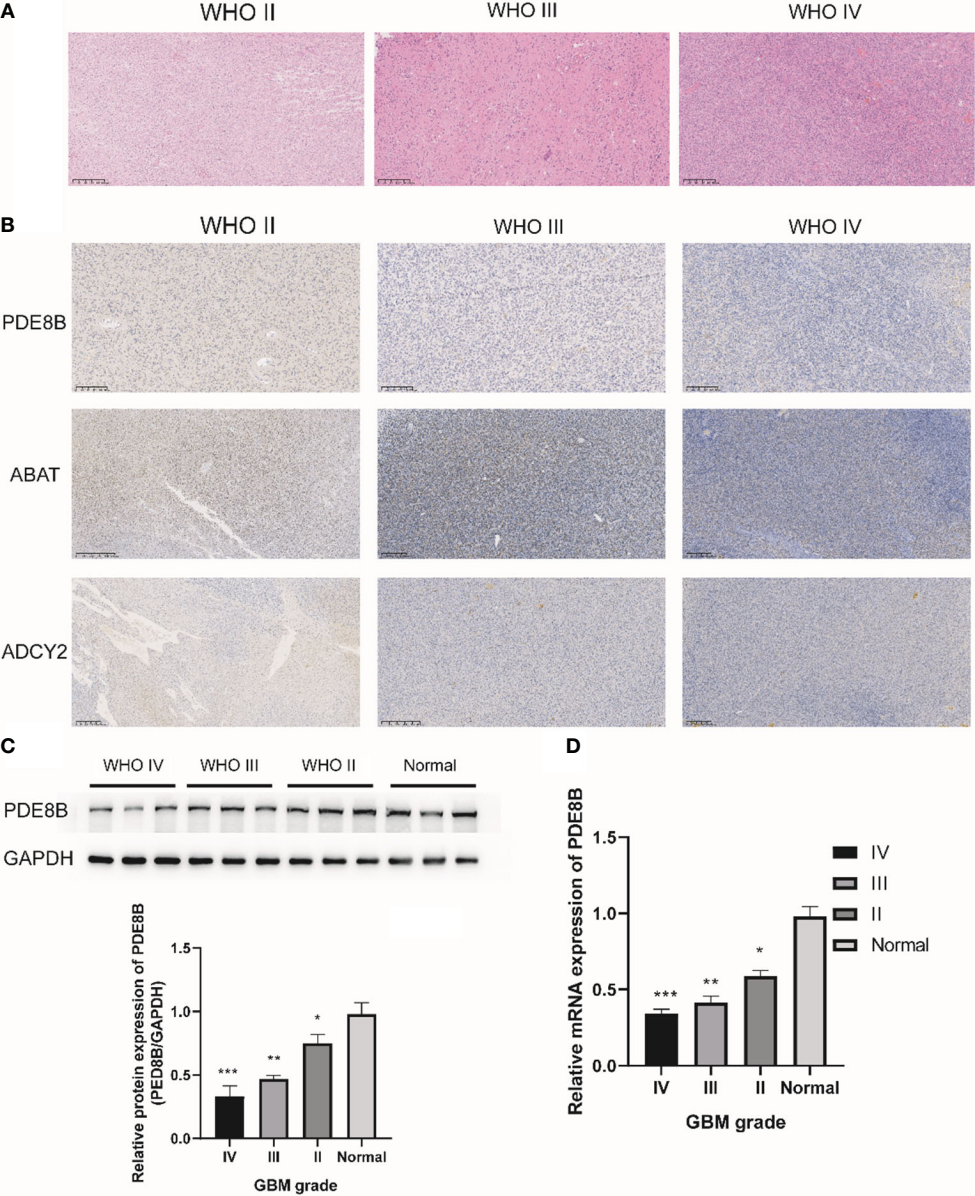
IHC staining for metabolic protein in glioma WHO II-IV. **(A)** H&E staining was used to detect the pathological characteristics of glioma WHO II-IV; **(B)** Representative images of IHC staining for PDE8B in glioma WHO II-IV; **(C)** Western blotting was used to detect the protein expression of PDE8B; **(D)** RT-qPCR assay was used to detect the mRNA expression of PDE8B. *P<0.05, **P<0.01, ***P<0.001.

H&E staining results indicated cytoplasmic and nuclear staining with mild atypia in glioma grade II; the density of glioma cells was moderately increased with varied sizes and disordered arrangement, and more pronounced atypia in glioma grade III; the density of glioma cells was significantly dense with mitotic figures and microvascular proliferation in glioma WHO grade IV (**Figure 7A**). Subsequent IHC staining revealed the expression of PDE8B, ABAT, and ADCY2 proteins in glioma WHO grades II-IV. The protein expression of PDE8B, ABAT, and ADCY2 decreased in glioma grade IV compared with glioma grades III and II. The expression of PDE8B, ABAT, and ADCY2 proteins decreased with increasing glioma WHO grade (**Figure 7B**). Further, Western blotting assay results suggested that the protein expression of PDE8B was markedly downregulated in glioma tissues compared with adjacent tissues (normal). The expression of PDE8B decreased with increasing glioma grade (**Figure 7C**). Moreover, RT-qPCR assay verified that the mRNA expression of PDE8B was significantly downregulated in the glioma group compared with the normal group. The expression of PDE8B decreased with the increase of glioma grade (II-IV) (**Figure 7D**).

## 4 Discussion

Low-grade glial neoplasms are among the most prevalent brain tumors in the pediatric population (56). High-grade gliomas, particularly glioblastoma (GBM), represent the most aggressive CNS cancers in adults (57). Due to its heterogeneous nature (40), glioblastomas nearly always recur post-surgery and after radio-/chemo-/immunotherapy treatments, making them highly lethal with a poor prognosis (58–60). The cellular origins of glioma remain a subject of intense debate (61). Various brain cell populations, including neural stem cells (NSCs) (4–7), astrocytes (9) and oligodendrocyte precursor cells (OPCs) (13, 62, 63), have been implicated in glioma development. For instance, Wang et al. developed a cell-lineage-based stratification model for glioblastoma, underscoring how the cell of origin shapes distinct molecular landscapes and therapeutic vulnerabilities even in the presence of identical driver mutations (64). The interaction of cell origins and oncogenic states influences glioma initiation and progression (65), affects the susceptibility to glioblastoma treatments (66), and determines whether tumors develop from astrocytes or oligodendroglial cells (67). Additionally, genetic alterations like IDH1/IDH2 mutations (68, 69) and epigenetic changes such as mutations in histone H3 genes (70), along with aberrant transcriptional activity (71, 72) and disrupted metabolism (16) intertwined with signaling pathways (73, 74), collaboratively impact the cellular origin, oncogenic state, tumor aggressiveness, and response to therapy (33, 75). In this study, we utilized single-cell and spatial transcriptomics to dissect the roles of cell origin, metabolic landscape, and transcriptional regulators in high-grade GBM and low-grade oligoastrocytoma, thereby enhancing our understanding of glioma’s cellular and molecular foundations.

Glioblastoma progression is closely linked to metabolic remodeling in both cancer and immune cells (19, 76–78), with aerobic glycolysis serving as the primary source of energy and biosynthetic precursors (79). Targeting the Warburg effect is now recognized as a promising therapeutic strategy (18, 80). Our study reveals distinct metabolic landscapes and pathway alterations between low-grade and high-grade gliomas, primarily within astrocytes, OPCs, and oligodendrocytes, aligning with histopathological findings (75). Beyond glycolysis/gluconeogenesis, we observed differential activities in amino acid pathways, such as phenylalanine metabolism, and lipid pathways, such as fatty acid biosynthesis and sphingolipid metabolism, suggesting a comprehensive metabolic disruption in glioma. Remarkably, we also noted metabolic pathway variations and gene expression differences in immune cells, particularly microglia, indicating that immune cell functions are also modulated by diverse metabolic reprogramming in cancer progression (81, 82). Future detailed investigations could elucidate the specific roles of these varied metabolic pathways and genes in glioma.

Metabolic pathway disturbances and gene disruptions are influenced by both extracellular and intracellular stresses (83, 84), leading to epigenetic and transcriptional modifications that drive cancer progression (85). Therefore, targeting metabolic pathways presents a viable therapeutic avenue (86, 87), though the clinical implications of these pathways in glioma require further exploration. Our research also assessed the clinical relevance and prognostic value of metabolic pathways and differential genes in glioma, identifying 48 prognostically significant metabolic pathways and 11 critical genes in OPCs and astrocytes. Among these, classical carbohydrate metabolism pathways like Glycolysis/Gluconeogenesis and the Pentose phosphate pathway showed detrimental effects, whereas the Citrate cycle (TCA cycle) exhibited beneficial effects, consistent with prior tumor studies (29, 88–90). The prognostic relevance of other metabolic pathways also warrants increased focus in glioma, highlighting both deleterious pathways like Purine metabolism and Glutathione metabolism and beneficial ones like Glycerolipid metabolism and Vitamin B6 metabolism. Furthermore, the 11 genes identified in astrocytes and OPCs displayed significant correlations with glioma grading, IDH mutation status, and overall prognosis, marking them as potential oncogenes or tumor suppressors and clinically relevant indicators for molecules like LDHA (91, 92), MIF (93, 94), and NAMPT (25, 95–98).

The cAMP/PKA signaling pathway serves as a pivotal regulator of metabolic pathways across various diseases, with the cAMP response finely tuned by phosphodiesterases (PDEs) (99). Abnormal expression of PDE8B has been linked to various diseases and cancer pathologies, including associations with metastasis in thyroid carcinoma (100). Intriguingly, we found that PDE8B is predominantly expressed in astrocytes and OPCs of glioma, as demonstrated through UMAP analysis. Tissue studies indicated that PDE8B expression decreases significantly in higher-grade GBM tissues compared to adjacent normal tissues, paralleling the progression to an immunosuppressed tumor microenvironment where both immune cells and PDE8B expression are diminished. Thus, PDE8B serves as a crucial biomarker in GBM, particularly within astrocytes and OPCs.

Moreover, our single-cell transcriptomic analysis revealed that PDE8B expression is markedly higher in low-grade than in high-grade gliomas, a finding substantiated at the cellular and tissue levels through various methods including qPCR, Western blotting, and immunohistochemistry. This observation challenges the traditional view that oncogenes are generally upregulated in tumor tissues to promote malignant behaviors. The underlying mechanisms suggest that while PDE8B expression is reduced in high-grade gliomas, it actively promotes tumor proliferation, invasion, and growth in high-grade glioblastoma cell lines, possibly due to differing cell origins in high-grade versus low-grade gliomas. Research indicates that high-grade gliomas might arise from malignant transformations of OPCs (37, 63, 101, 102), while low-grade gliomas typically develop from astrocytes and oligodendrocytes (54, 103, 104). This suggests that PDE8B may perform divergent, possibly even contradictory roles depending on the specific type of tumor cell, akin to recent findings that miRNAs, although highly expressed, can suppress tumor growth (105). Additionally, the structural and functional similarities between PDE8B and another family member, PDE8A, which has been found to regulate stemness in glioma-initiating cells and exhibit tumor-suppressive properties (106), further complicate the understanding of their roles in cancer. Indeed, inhibiting PDE8A in melanoma has been shown to suppress the MAPK pathway and tumor growth, hinting at an oncogenic role for PDE8A (107). This complexity underscores the need for a deeper exploration of the non-canonical functions of these enzymes, as exemplified by the metabolic enzyme LDHA, which has been shown to activate Rac1 GTPase through non-traditional mechanisms to promote cancer (108). Similarly, aside from its established role in cAMP hydrolysis, PDE8B may engage in non-canonical activities that could elucidate its function in glioma progression.

Here, the predictive role of PDE8B as an important biomarker in GBM is highlighted, especially in astrocytes and OPCs. However, the functional characteristics of PDE8B are not clear, and the specific molecular mechanism and pathway of PDE8B when it plays its role have not been solved, it needs to be further studied with animal models or cell models, which is also one of the limitations. At the same time, in addition to PDE8B, a large number of other genes were also mentioned in this article, but we did not fully and completely discuss or verify them, which is a pity, and we hope that this study will provide some ideas and references for readers.

In summary, our study comprehensively investigates the origins, metabolic profiles, and clinical implications of gliomas across different grades, highlighting the significant role of the metabolism-associated enzyme PDE8B as both a biomarker and a driver of glioma progression. This research not only advances our understanding of the complex functions of PDE8B in gliomas but also suggests new avenues for targeted therapeutic strategies.

## Data availability statement

The data presented in the study are deposited in the NCBI repository, accession number GSE270109.

## Ethics statement

This study was approved by the ethical committee of Sichuan Academy of Medical Sciences and Sichuan Provincial People’s Hospital (2023–457). The studies were conducted in accordance with the local legislation and institutional requirements. Written informed consent for participation in this study was provided by the participants’ legal guardians/next of kin.

## Author contributions

ZH: Data curation, Writing – original draft. YP: Data curation, Methodology, Writing – original draft. DW: Methodology, Writing – original draft. CY: Writing – original draft. CZ: Writing – original draft. BG: Funding acquisition, Writing – review & editing. SS: Project administration, Supervision, Validation, Writing – review & editing. YW: Funding acquisition, Investigation, Supervision, Validation, Writing – review & editing.

## Supplementary material

Supplementary Figure 1 Overall metabolism pathway activities in glioma samples with clinical parameters. **(A)** Overall metabolism pathway activities in glioma samples with clinical parameters, in TCGA datasets; **(B)** Overall metabolism pathway activities in glioma samples with clinical parameters, in CGGA datasets; **(C)** Differential metabolism pathway activities of pyruvate metabolism were shown in TCGA and CGGA datasets.

Supplementary Figure 2 Kaplan-Meier survival plot of representative 12 metabolism pathways in TCGA and CGGA.

Supplementary Figure 3 TSNE map showing the expression of 11 metabolism genes.

Supplementary Figure 4 Kaplan-Meier survival plot of representative 11 metabolism genes and HIF1A in CGGA **(A)** and TCGA **(B)**, in all glioma samples.

Supplementary Figure 5 Kaplan-Meier survival plot of representative 11 metabolism genes in CGGA **(A)** and TCGA **(B)**, in high grade and low–grade glioma samples.

Supplementary Table 1 All 79 metabolic changed pathways (MCPs) in HG and LG.

Supplementary Table 2 All 35 most changed MCPs in 9 cell types.

Supplementary Table 3 Clinically significant pathways in CGGA and TCGA datasets.

Supplementary Table 4 Prognostic 48 significant pathways.

Supplementary Table 5 Differential expression genes (DEGs) in 10 cell types.
