# Supplementary figures and images for: Single-cell transcriptomic analysis identifies downregulated phosphodiesterase 8B as a novel oncogene in IDH-mutant glioma
Source: Front Immunol. 2024 Jun 26;15:1427200. doi: 10.3389/fimmu.2024.1427200 (PMC11233524; doi:10.3389/fimmu.2024.1427200)

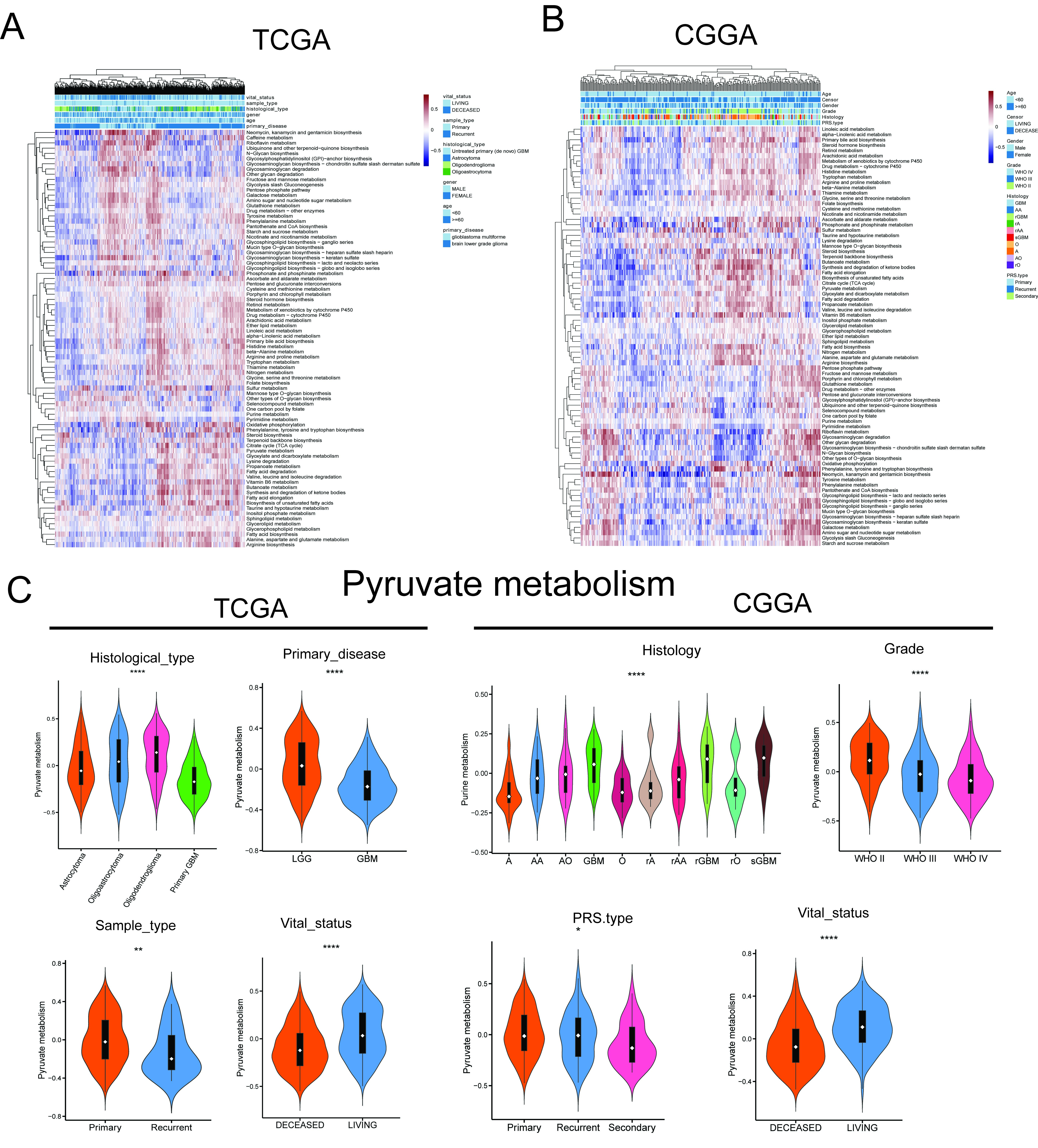

Supplement: Supplementary Figure 1 — Overall metabolism pathway activities in glioma samples with clinical parameters. (A) Overall metabolism pathway activities in glioma samples with clinical parameters, in TCGA datasets; (B) Overall metabolism pathway activities in glioma samples with clinical parameters, in CGGA datasets; (C) Differential metabolism pathway activities of pyruvate metabolism were shown in TCGA and CGGA datasets. [file Image_1.jpeg]

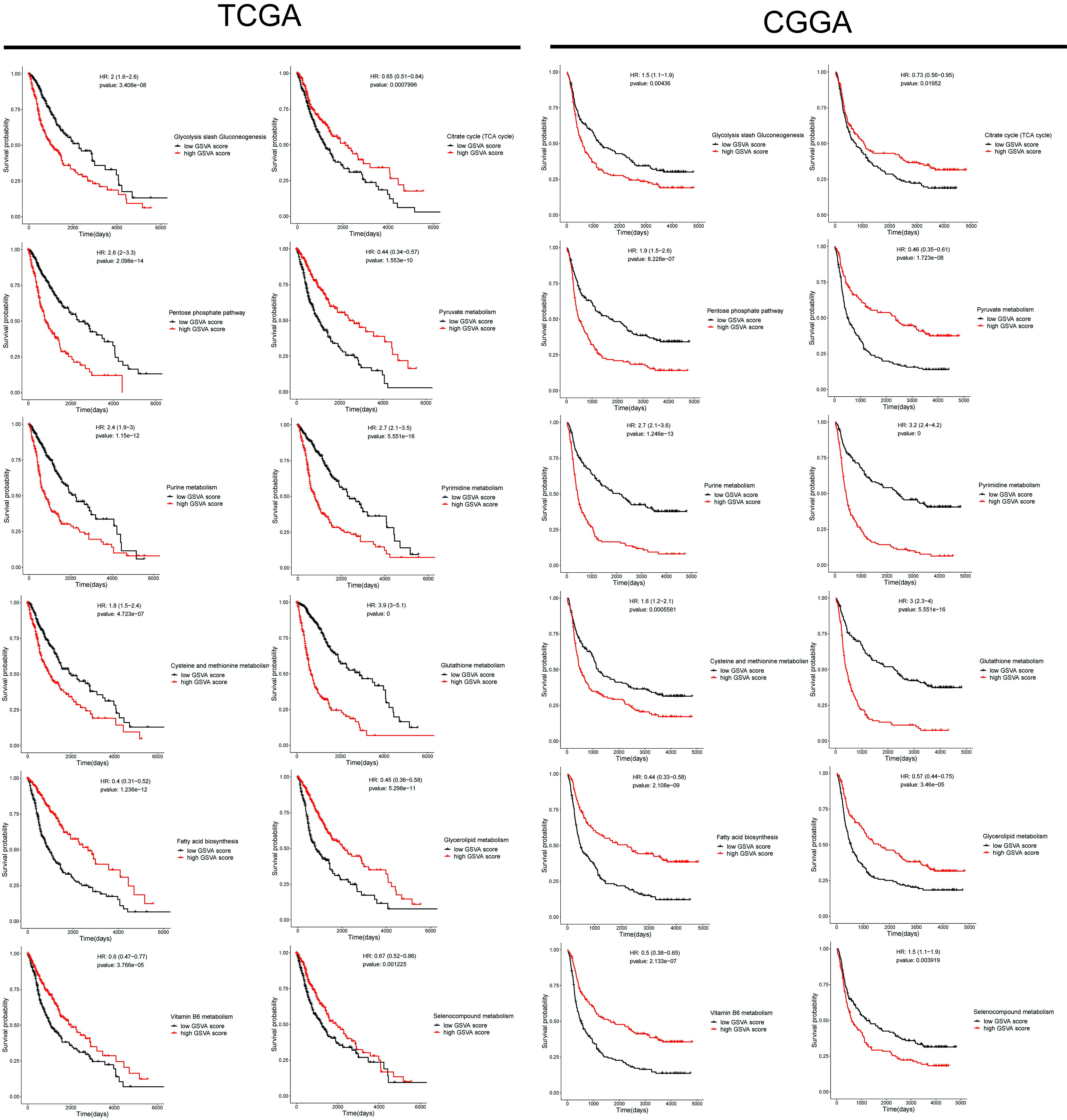

Supplement: Supplementary Figure 2 — Kaplan-Meier survival plot of representative 12 metabolism pathways in TCGA and CGGA. [file Image_2.jpeg]

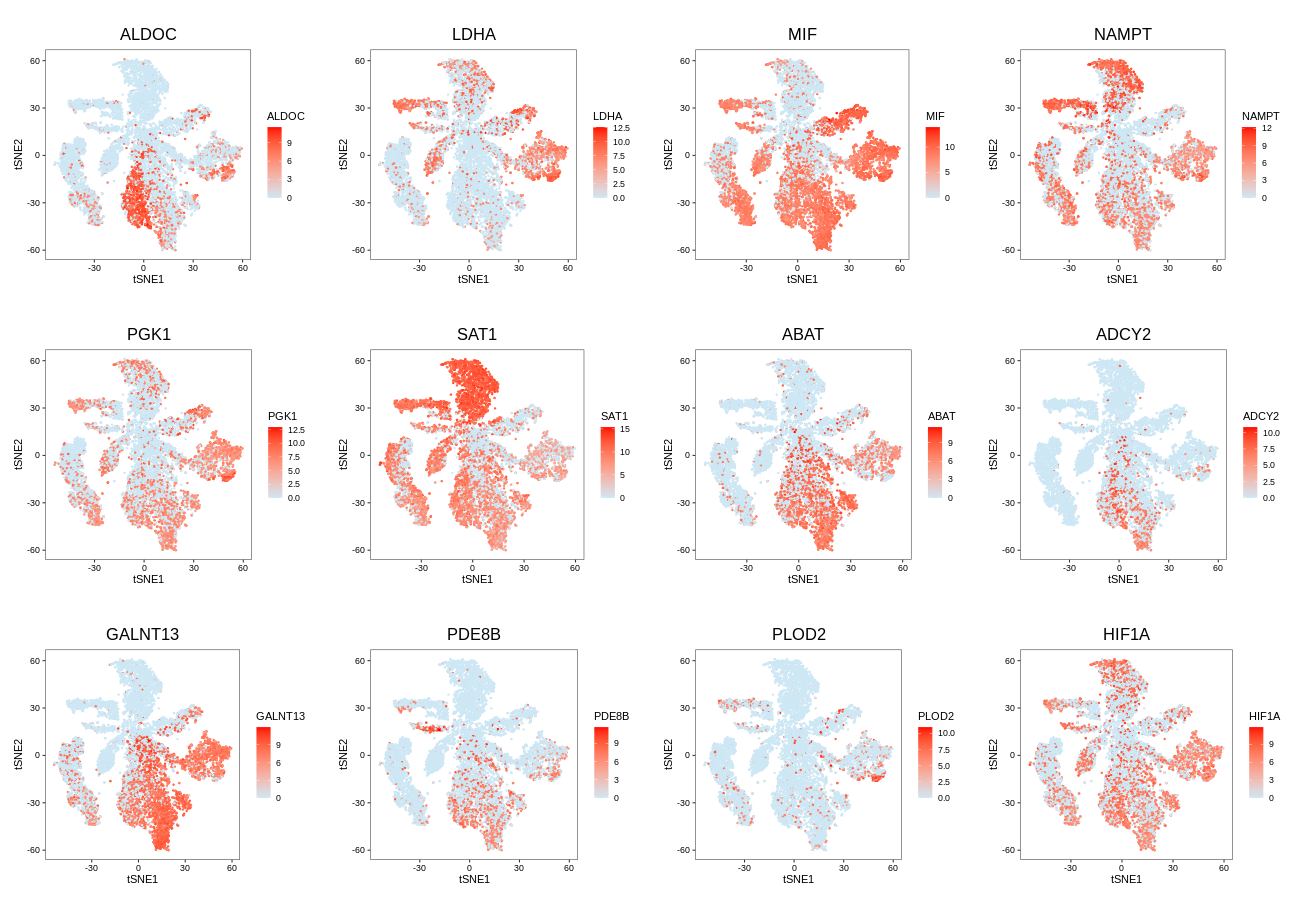

Supplement: Supplementary Figure 3 — TSNE map showing the expression of 11 metabolism genes. [file Image_3.tif]

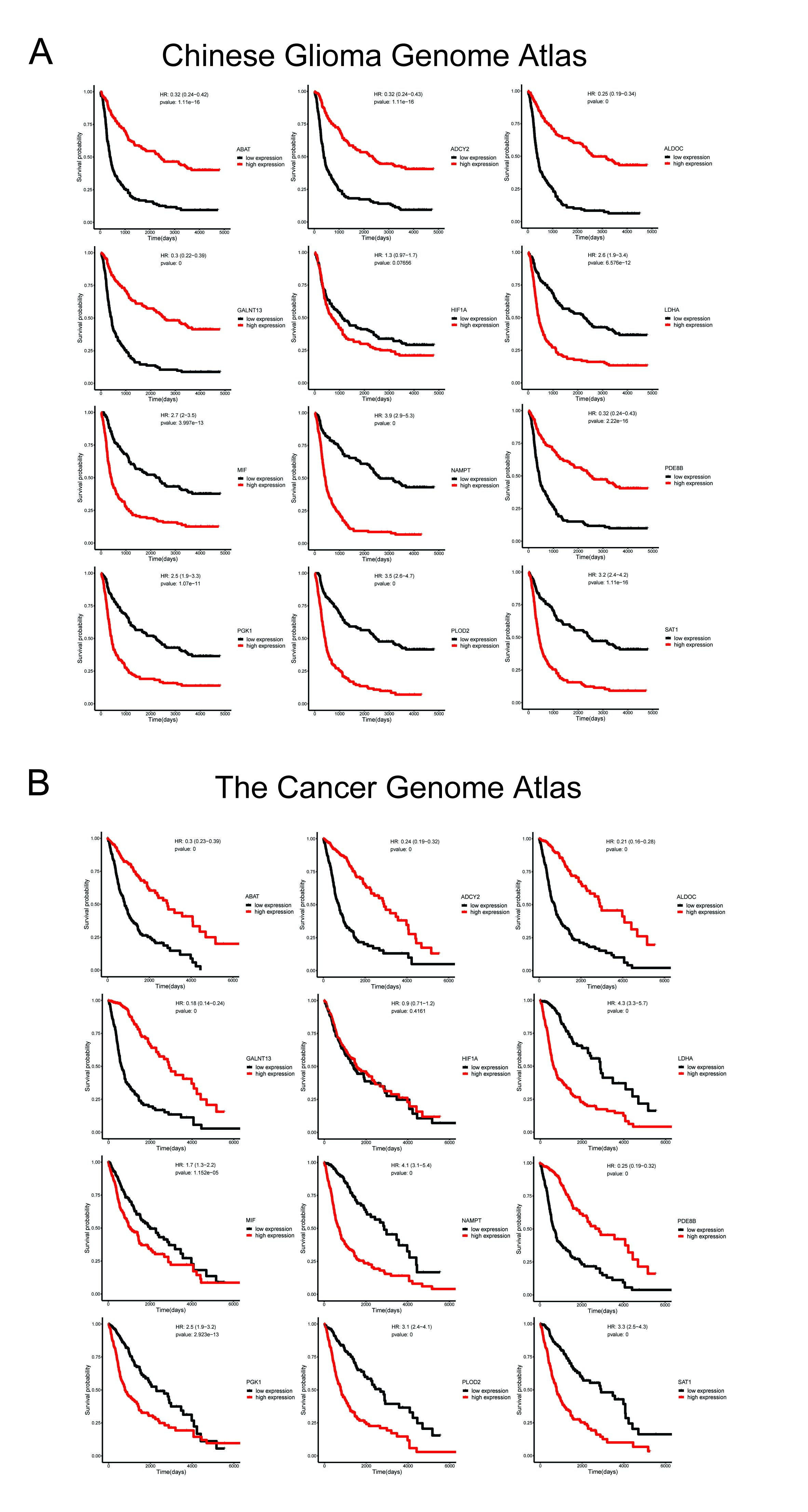

Supplement: Supplementary Figure 4 — Kaplan-Meier survival plot of representative 11 metabolism genes and HIF1A in CGGA (A) and TCGA (B), in all glioma samples. [file Image_4.jpeg]

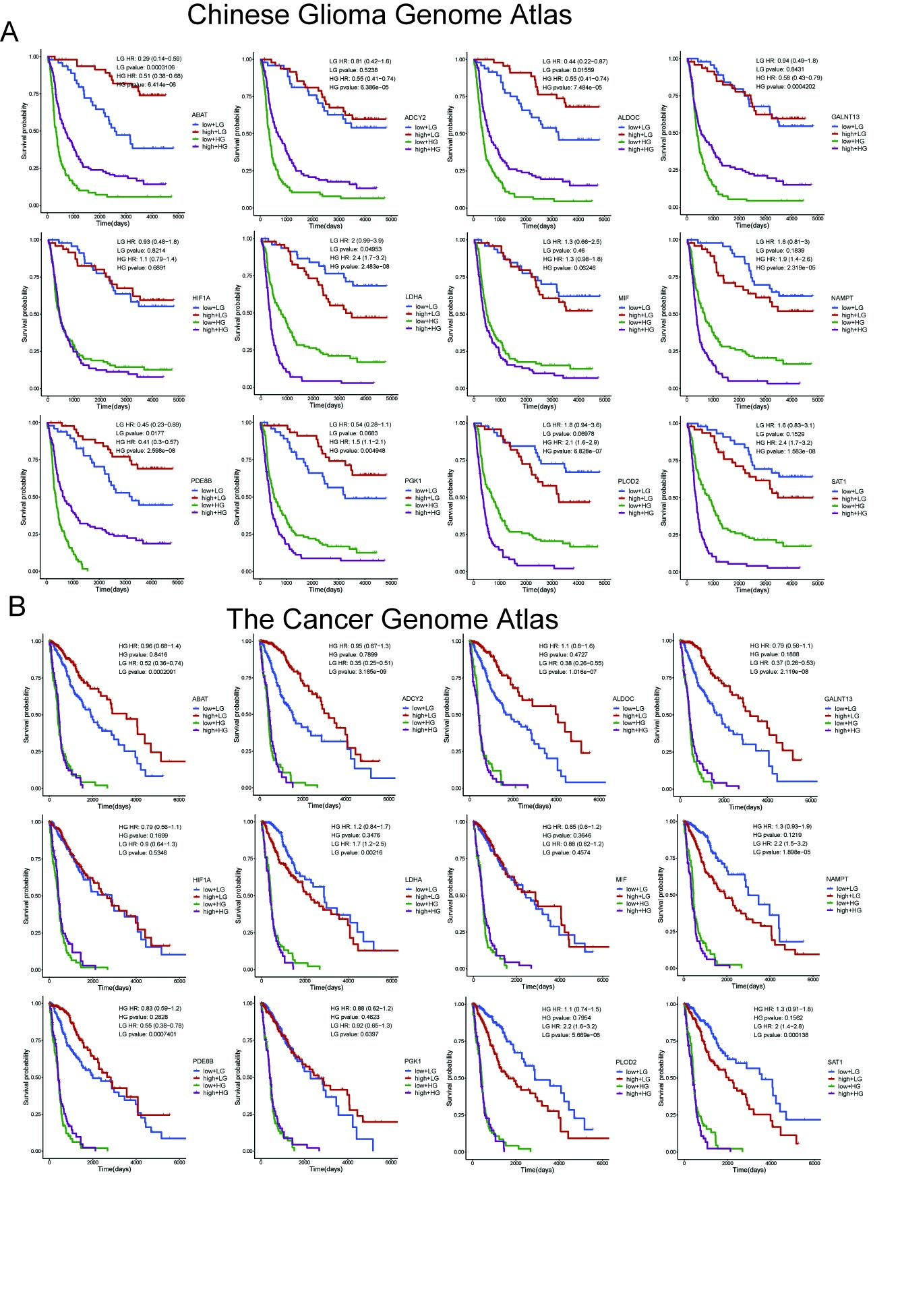

Supplement: Supplementary Figure 5 — Kaplan-Meier survival plot of representative 11 metabolism genes in CGGA (A) and TCGA (B), in high grade and low–grade glioma samples. [file Image_5.jpeg]
